# A Modified MinMax *k*-Means Algorithm Based on PSO

**DOI:** 10.1155/2016/4606384

**Published:** 2016-08-30

**Authors:** Xiaoyan Wang, Yanping Bai

**Affiliations:** ^1^School of Information and Communication Engineering, North University of China, Taiyuan 030051, China; ^2^School of Science, North University of China, Taiyuan 030051, China

## Abstract

The MinMax *k*-means algorithm is widely used to tackle the effect of bad initialization by minimizing the maximum intraclustering errors. Two parameters, including the exponent parameter and memory parameter, are involved in the executive process. Since different parameters have different clustering errors, it is crucial to choose appropriate parameters. In the original algorithm, a practical framework is given. Such framework extends the MinMax *k*-means to automatically adapt the exponent parameter to the data set. It has been believed that if the maximum exponent parameter has been set, then the programme can reach the lowest intraclustering errors. However, our experiments show that this is not always correct. In this paper, we modified the MinMax *k*-means algorithm by PSO to determine the proper values of parameters which can subject the algorithm to attain the lowest clustering errors. The proposed clustering method is tested on some favorite data sets in several different initial situations and is compared to the *k*-means algorithm and the original MinMax *k*-means algorithm. The experimental results indicate that our proposed algorithm can reach the lowest clustering errors automatically.

## 1. Introduction

Clustering has broad applications in pattern recognition, image processing, machine learning, and statistics [1, 2]. The aim is to partition a collection of patterns into disjoint clusters, such that patterns in the same cluster are similar, and, on the other hand, patterns of two different clusters are distinct.

One of the most popular clustering methods is *k*-means algorithm, where clusters are identified by minimizing the clustering error. The *k*-means algorithm is widely accepted in the literature. However, the *k*-means algorithm is sensitive to the choice of initial starting conditions [3, 4].

To deal with this problem, several methods have been proposed. For example, a method has been proposed to eliminate the dependence on random initial conditions. Global *k*-means algorithm [5] is an incremental approach that starts from one cluster and at each step a new cluster is deterministically added to the solution according to an appropriate criterion. Based on the algorithm, Bagirov et al. proposed some modifications [6, 7]. Tzortzis and Likas extended the algorithm to kernel space [8, 9]. Zang et al. developed a fuzzy *c*-means clustering algorithm and applied such algorithm to the investigation of speech signal [10]. An alternative approach to eliminate the influence of initial starting conditions is to use the multi-restarting *k*-means algorithm [11–14]. A new version of this method is the MinMax *k*-means clustering algorithm [15], which starts from a randomly picked set of cluster centers and tries to minimize the maximum intraclustering error. Its application [16] shows that the algorithm is efficient in intrusion detection.

Particle Swarm Optimization (PSO), a population-based stochastic search process, was firstly proposed by Eberhart and Kennedy in 1995 [17]. Such process was introduced to simulate the social behaviors of bird flocking or fish schooling when the group of birds or fish searches for food. PSO is very fast, simple, and easy to understand and implement. PSO does not require the adjustment of many parameters and the memory space required by the algorithm is little. PSO has been widely used to improve other algorithms' performances such as ANN [18–20], scheduling problems [21, 22], traveling salesman problems [23, 24], and anomaly detection problems [25]. PSO also has been successfully applied in clustering problem [26–30]. Recently, PSO and *k*-means algorithm are combined to develop novel clustering algorithms [31, 32].

In this paper, a new version of modified MinMax *k*-means algorithm is proposed. Recent investigation indicates that if the parameter of *p*_max_ has been set, the programme can reach the lowest *E*_max_ at *p* ∈ [*p*_init_, *p*_max_] [15]. However, experiments imply that the above conclusion is not always correct.

Different parameters of *p*_max_ result in different values of *E*_max_, and as such the value does not always comply with the rule that the larger the value of *p*_max_, the lower the value of *E*_max_. In MinMax *k*-means algorithm, parameter *β* needs priory set as well, and different values of *β* also result in different values of clustering errors. The value of clustering errors does not have any regulation. Therefore we should decide the values of parameters to minimize cluster errors.

In this paper, we calculate the clustering errors for each parameter *p*, respectively, without using the automatically adapted exponent *p* as in [15]. By utilizing PSO, we choose the parametric value and obtain the minimum clustering errors. Thus, we can obtain the minimum clustering errors without choosing parameters manually.

We carry out many experiments on different data sets, including synthetic data sets and real world data sets in five different initial situations. Balanced type, unbalanced type, and almost balanced type data sets are considered in the study. Our investigation indicates that the proposed algorithm can search proper parameters to get the minimum clustering errors.

The rest of the paper is organized as follows. We briefly describe the *k*-means, MinMax *k*-means, and PSO algorithms in Section 2. In Section 3 we propose our algorithms. Experimental evaluation is presented in Section 4. Lastly, Section 5 concludes our work.

## 2. Preliminaries

### 2.1. *k*-Means Algorithm

Given a data set *X* = {*x*_1_, *x*_2_,…, *x*_*N*_}, *x*_*n*_ ∈ *R* ^*d*^  (*n* = 1,2,…, *N*), we aim to partition this data set into *M* disjoint clusters *C*_1_, *C*_2_,…, *C*_*M*_, such that a clustering criterion is optimized. Usually, the clustering criterion is the sum of the squared Euclidean distances between each data point *x*_*n*_ and the corresponding cluster center *m*_*k*_. This kind of criterion is called clustering error and depends on the cluster centers *m*_1_, *m*_2_,…, *m*_*k*_:

(1) $$\begin{array}{c} E\left(\;m_{1},m_{2},\ldots ,m_{M}\;\right)=\overset{N}{\underset{i=1\;\;}{\sum }}\overset{M}{\underset{k=1}{\sum }}I\left(\;x_{i}\in C_{k}\;\right){\left\|\;x_{i}-m_{k}\;\right\|}^{2}, \end{array}$$

where

(2) $$\begin{array}{c} I\left(\;X\;\right)=\left\{\begin{array}{cc} 1, & X\text{ is true},\\ 0, & \text{otherwise}. \end{array}\right. \end{array}$$

Generally, we call ∑_*k*=1_^*M*^ *I*(*x*_*i*_ ∈ *C*_*k*_)‖*x*_*i*_ − *m*_*k*_‖^2^ intraclustering error (variance). Obviously, clustering error is the sum of intraclustering error. Therefore, we use *E*_sum_ to denote *E*(*m*_1_, *m*_2_,…, *m*_*M*_); that is, *E*_sum_ = *E*(*m*_1_, *m*_2_,…, *m*_*M*_).

The *k*-means algorithm finds locally optimal solutions with respect to the clustering error. The main disadvantage of the method is its sensitivity to initial position of the cluster center.

### 2.2. The MinMax *k*-Means Algorithm

As is known, in the *k*-means algorithm, we minimize the clustering error. The MinMax *k*-means algorithm minimizes the maximum intraclustering error:

(3) $$\begin{array}{c} E_{max}=\underset{1\leq k\leq M}{\max}\overset{N}{\underset{i=1}{\sum }}I\left(\;x_{i}\in C_{k}\;\right){\left\|\;x_{i}-m_{k}\;\right\|}^{2}, \end{array}$$

where *m*_*k*_, *I*(*x*) are defined as (1).

Since directly minimizing the maximum intracluster variance *E*_max_ is difficult, a relaxed maximum variance objective was proposed [15]. The authors constructed a weighted formulation *E*_*w*_ of the sum of the intracluster variances:

(4) Ew=∑k=1Mwkp∑i=1NIxi∈Ckxi−mk2,wk≥0,  ∑k=1Mwk=1,  0≤p≤1,

where the *p* exponent is a constant. The greater (smaller) the *p* value is, the less (more) similar the weight values become, as relative differences of the variances among the clusters are enhanced (suppressed).

In [15], the authors give a practical framework that extends the MinMax *k*-means to automatically adapt the exponent *p* to the data set. It begins with a small *p* (*p*_init_) that is increased by *p*_step_ after each iteration, until a maximum value *p* (*p*_max_) is attained. For the method, we should first decide the values of parameters *p*_init_, *p*_max_, and *p*_step_.

Now, all clusters contribute to the objective, according to different degrees regulated by the *w*_*k*_ values. It is clear that the more a cluster contributes (higher weight), the more intensely its variance will be minimized. So *w*_*k*_ are calculated by

(5) wk=vk1/1−p∑k′=1Mvk′1/1−p,where  vk=∑i=1NIxi∈Ckxi−mk2.

To enhance the stability of the MinMax *k*-means algorithm, a memory effect could be added to the weights:

(6) wkt=βwkt−1+1−βvk1/1−p∑k′=1Mvk′1/1−p,0≤β≤1.

### 2.3. PSO

PSO is a population-based metaheuristic algorithm. It is launched with a population (called a swarm) of individuals (called particles), where each particle represents a potential solution in the problem space. All of the particles move around the problem space to find the optimization solution (the best position) according to some speed (velocity) iteratively. In the *n*-dimension problem space, the position and the velocity of the *i*th particle are, respectively, denoted by the following vectors:

(7) Xit=xi1t,xi2t,…,xint,Vit=vi1t,vi2t,…,vint.

The solution is evaluated by the fitness value for each particle at every iteration. Afterwards, a record of the best position of each particle based on fitness value is saved. The best previously visited position of the particle *i* at iteration *t* is denoted by vector *P*_*i*_(*t*) = (*p*_*i*1_(*t*), *p*_*i*2_(*t*),…, *p*_*in*_(*t*)) as the personal best. The position of all the particles which give the best fitness value at iteration *t* is also recorded as the global best position denoted by *G*(*t*) = (*g*_1_(*t*), *g*_2_(*t*),…, *g*_*n*_(*t*)).

At each iteration, the velocity and the position of each particle are updated according to the following equations:

(8) Vit=ω·Vit−1+c1·r1·Pit−1−Xit−1+c2·r2·Gt−1−Xit−1,Xit=Xit−1+Vit,

where *ω* is an inertia weight that introduces a preference for the particle to continue moving in the same direction. Here, *c*_1_ and *c*_2_ are two positive constant parameters called coefficients, and *r*_1_ and *r*_2_ denote two random numbers between (0,1). In order to prevent the particle's blind search, each component of *V*_*i*_ and each component of *X*_*i*_ are kept within the ranges [−*V*_max_, *V*_max_] and [−*X*_max_, *X*_max_], respectively.

Inertia weight is not included in the original version [17]. The inclusion of an inertia weight in the PSO was first proposed by Shi and Eberhart [33]; then they subsequently investigated the effect of the inertial weight and maximum velocity on the performance of the particle swarm optimizer, and they provided guidelines for selecting these two parameters [34]. There are several strategies of inertial weight *ω* described in [20, 35–37], and there are some other ways to insure the convergence of the PSO, that is, a constriction factor [38] and a conditional random [39].

## 3. The Proposed Algorithm

The effectiveness and robustness of the MinMax *k*-means algorithm depend on initializations of parameters [15]. Reference [15] introduces a practical framework that extends the MinMax *k*-means to automatically adapt the exponent *p* to the data set. They concluded that if *p*_max_ has been set, the programme can reach the lowest *E*_max_ at *p* ∈ [*p*_init_, *p*_max_]. However, our experiments show that it is not always correct. We do experiments using the well-known data set Pendigits to support our claim. The description of this data set is given in Section 4. In our calculations the results of the MinMax *k*-means algorithm are the average over 100 runs of *E*_max_ and *E*_sum_ defined by (3) and (1), respectively. The results are reported in Table 1. One can see from Table 1 that *E*_max_ and *E*_sum_ have different values for different *p* and *β*. To address this problem we propose a new algorithm to find optimal values of parameters *p* and *β* which provide the minimum values of *E*_max_ and *E*_sum_. We call it the PSO MinMax *k*-means algorithm.

The proposed algorithm includes the PSO process and the MinMax *k*-means process. We utilize PSO to optimize the two parameters. That is to say, we find optimal parameters and put them into MinMax *k*-means process to obtain the minimum clustering errors. The specific method is illustrated as follows.

**Algorithm 1 (PSO MinMax *k*-means algorithm).**

See Figure 1.

*Step 1*. Set up parameters of PSO, including iteration, population size, maximum velocity (*V*_max_), inertial weight (*ω*), and two learning factors (*c*_1_, *c*_2_); give the number of clusters *k* and initial weight *w* = 1/*k*.

*Step 2*. Initialize each particle position (*X*_*i*_) and velocity (*V*_*i*_) randomly; randomly choose the center of each cluster. Note that each particle is a vector of the two parameters (*p*, *β*) for MinMax *k*-means algorithm. Therefore, *X*_*i*_ can be represented as *X*_*i*_ = (*p*_*i*_, *β*_*i*_).

*Step 3*. Calculate cluster assignments *I*(*x*_*i*_ ∈ *C*_*j*_), *i* = 1,2,…, *N*, by the Euclidean distance for each particle.

*Step 4*. Calculate the weighted sum of the intracluster variances *E*_*w*_ by (4) for each particle.

*Step 5*. Update cluster center *C*_*j*_, *j* = 1,2,…, *k*, by the following equation: *m*_*j*_ = (1/|*C*_*i*_|)∑_*x*_*i*_∈*C*_*j*__ *x*_*i*_ for each particle.

*Step 6*. Update weight value by (6).

*Step 7*. If stopping conditions of MinMax *k*-means algorithm are not satisfied, go back to Step 3; otherwise, go to Step 8.

*Step 8*. Calculate fitness value for each particle using formula (1) or (3). That is to say, clustering errors are the fitness functions.

*Step 9*. Update the personal best *P*_*i*_ and the global best *G*.

*Step 10*. According to the best positions *P*_*i*_ and *G*, update the velocity and position for each particle using formula (8). It should be noted that *V* cannot be larger than *V*_max_ or smaller than −*V*_max_, and *X* cannot be larger than *X*_max_ or smaller than −*X*_max_. Thus,

(9) Vij=Vmax,if  Vij>Vmax,Vij,if  Vmax>Vij>−Vmax,−Vmax,if  −Vmax>Vij,Xij=Xmax,if  Xij>Xmax,Xij,if  Xmax>Xij>−Xmax,−Xmax,if  −Xmax>Xij.

*Step 11*. Record the final best *p* and *β* if the specified number of iterations is reached; otherwise, go back to Step 8.

After performing all the steps above, we find the optimal parameters of MinMax *k*-means algorithm and then get the final clustering results by plugging the optimal parameter to MinMax *k*-means algorithm. Actually Steps 3 to 7 are the process of MinMax *k*-means algorithm.

## 4. Computational Results

In the following subsections, we report experimental results for five different states (State 1 to State 5) using both synthetic and real world data sets. We also compare the *k*-means, the MinMax *k*-means, and the PSO MinMax *k*-means algorithms using numerical results.

In each state, the results of the MinMax *k*-means algorithm are tested in different parameters (*p*_max_, *β*), and we set *p*_init_ = 0, *p*_step_ = 0.01 as in [15]; we set the population size 20 and the generation number 100 in PSO MinMax *k*-means algorithm. The experiments in each state of PSO MinMax *k*-means algorithm just have two sets of results. The value of parameters in each set is one of the optimization values which can result in one minimum of the clustering errors.

### 4.1. Synthetic Data Sets

**Figure 2:**
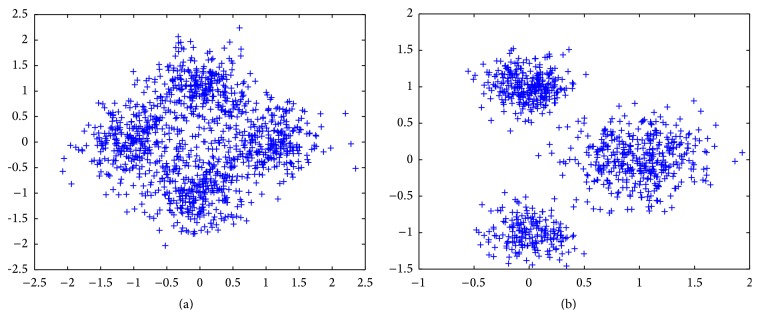
The sketch of two typical synthetic data sets: (a) *S*_1_ and (b) *S*_2_.

In this subsection, two synthetic data sets *S*_1_ and *S*_2_ from [40] are used to test algorithms. Typically, they are generated from a mixture of four or three bivariate Gaussian distributions on the plane coordinate system. Thus, a cluster takes the form of a Gaussian distribution. Particularly, all the Gaussian distributions have the covariance matrices of the form *σ* ^2^ *I*, where *σ* is the standard deviation. The first data set *S*_1_ with four Gaussian distributions and 300 sample points is located at (−1,0), (1,0), (0,1), and (0, −1), respectively. Actually, *σ* takes the values of 0.4. As for data set *S*_2_, we give three Gaussian distributions located at (1,0), (0,1), and (0, −1), with 400, 300, and 200 sample points, respectively. Therefore, *S*_2_ represents the asymmetric situation where the clusters do not take the same shape with different number of sample points. The data sets are shown in Figure 2, respectively.

### 4.2. Real World Data Sets

Coil-20 is a data set [41], which contains 72 images taken from different angles for each of the 20 included objects. We use three subsets, Coil15, Coil18, and Coil19, with images from 15, 18, and 19 objects, respectively, as the data set in [15]. The data set includes 216 instances and each of the data instances has 1000 features.

Yeast (UCI) [42] includes 1484 instances about the cellular localization sites of proteins and eight attributes. Proteins belong to ten categories. Five of the classes are extremely underrepresented and are not considered in our evaluation. The data set is unbalanced.

Pendigits (UCI) [42] includes 10992 instances of handwritten digits (0–9) from the UCI repository [19] and 16 attributes. The data set is almost balanced. In the experiment, the sample data of Pendigits data set will be firstly normalized and then the algorithm will be implemented on the normalized data.

Ecoli (UCI) [42] is composed of 336 protein localization sites for the* E. coli* bacterium and seven attributes. Proteins belong to eight different categories. Four of the classes are extremely underrepresented and are not considered in our evaluation. The data set is unbalanced.

A summary of the data sets is provided in Table 4.

### 4.3. Performance Analysis

The comparison of the algorithms across the various data sets in five different states is shown in Tables 2, 3, and 5 [Table tab6] [Table tab7]–8. The values of parameters for PSO MinMax algorithm in Tables 2, 3, and 5 [Table tab6] [Table tab7]–8 are shown in Table 9 correspondingly. Based on the analysis shown in the tables, first, we find that our proposed algorithm can attain the lowest *E*_max_ and *E*_sum_ values in optimal parameters except in Table 5 (States 2–5). In States 2–5 of Table 5, our proposed algorithm did not attain the lowest *E*_max_. It lies in the drawbacks of PSO algorithm itself which just gets the local optimal solution. Sometimes our proposed algorithm has better *E*_max_ than *k*-means algorithm and the original MinMax *k*-means algorithm (see Tables 3 and 7) and sometimes we have both better *E*_max_ and *E*_sum_ than other algorithms. We also find that *E*_max_ and *E*_sum_ cannot reach the lowest value simultaneously.

Second, it follows from the tables that the values of clustering errors in *k*-means and in MinMax *k*-means are equal when setting *p*_max_ = 0, *β* = 0, implying that the *k*-means algorithm can be considered as a special case of the MinMax *k*-means algorithm.

Third, the proper parameter in our algorithm is not a single value. Here, we just give one of the values. Hence, MinMax *k*-means algorithm and our proposed algorithm can both reach the lowest clustering errors on different parameter value.

Fourth, for the operation time of the algorithm, it is easy to observe that *k*-means algorithm consumes the least time. The operation time of our proposed algorithm depends on the population size (*s*), the number of generation (*n*), and the speed of convergence. For convenience, denote the running time of single MinMax *k*-means algorithm as *t*; then the operation time of our proposed algorithm is *snt*. Comparing the running time of MinMax *k*-means to that of our proposed algorithm, it is hard to identify which method consumes less time. For example, when we perform Coil2 data set (State 1) with *p*_max_ = 0.5, *β* = 0.3, the running time of MinMax *k*-means is 0.7403 s, and the running time of our proposed algorithm (*s* = 3, *n* = 2) is 0.6653 s. However, when we perform experiment on Ecoli data set (State 1), the running time is 0.3637 s for MinMax *k*-means (*p*_max_ = 0.5, *β* = 0.3) and the running time for our proposed algorithm (*s* = 10, *n* = 5) is 0.7848 s.

Finally, Tzortzis and Likas [15] stated that high *p* value forces clusters with large variance to lose most or even all of their instances as their enormous weights excessively distance the instances from their centers.

Hence, their solution has the following properties: whenever an empty or singleton cluster emerges, no matter if *p*_max_ has been reached or not, decreasing *p* by step reverts back to the cluster assignments corresponding to the previous *p* value and clustering must be resumed from there. Our algorithm can resolve the problem automatically. We do experiment on all data sets mentioned in this paper. The result is as follows. On Ecoli data set (State 5) when *p*_max_ = 0.5, *β* = 0, MinMax *k*-means converges for *p* = 0.44 with *E*_max_ = 5.4347, *E*_sum_ = 16.1918. On the other hand, if *p* ≥ 0.45, *β* = 0, we have *E*_max_ = 49.9010, *E*_sum_ = 49.9010. It is clear that when *p* is bigger than convergence value, the corresponding values of *E*_max_ and *E*_sum_ are much bigger than that of convergence value. The situations on States 1–4 are similar to State 5. On Pendigits data set, when *β* = 0, MinMax *k*-means algorithm converges for *p* < 0.5. If *p* is bigger than the convergence value, we get *E*_max_ = *E*_sum_, which is much bigger than that of proper *p*. Similar situation occurs on Yeast data set. In summary, we find that when *p* is bigger than the convergence value, we get *E*_max_ = *E*_sum_, which are much bigger than that of proper *p*; meanwhile the data sets are clustered just for one class. Based on the above analysis, our proposed algorithm cannot choose the corresponding parameter in PSO process. Therefore our proposed algorithm cannot be chosen singleton or empty for one of the clusters.

## 5. Conclusions

We modified the MinMax *k*-means algorithm to attain the lowest clustering errors automatically. Firstly, we use PSO to search the optimal parameters which can result in the minimum errors. Then we plug the parameters obtained by PSO into MinMax *k*-means algorithm. Experiments are tested on different data sets in different initial states, and the results show that our proposed algorithm is efficient in most situations.

As for future work, we plan to accelerate the proposed algorithm. A possible direction is data sets processing. For example, we can use the method of PCA. We also plan to achieve time efficiency of the PSO process, since many iterations in the algorithm may have repetitive calculations.

## Figures and Tables

**Figure 1:**
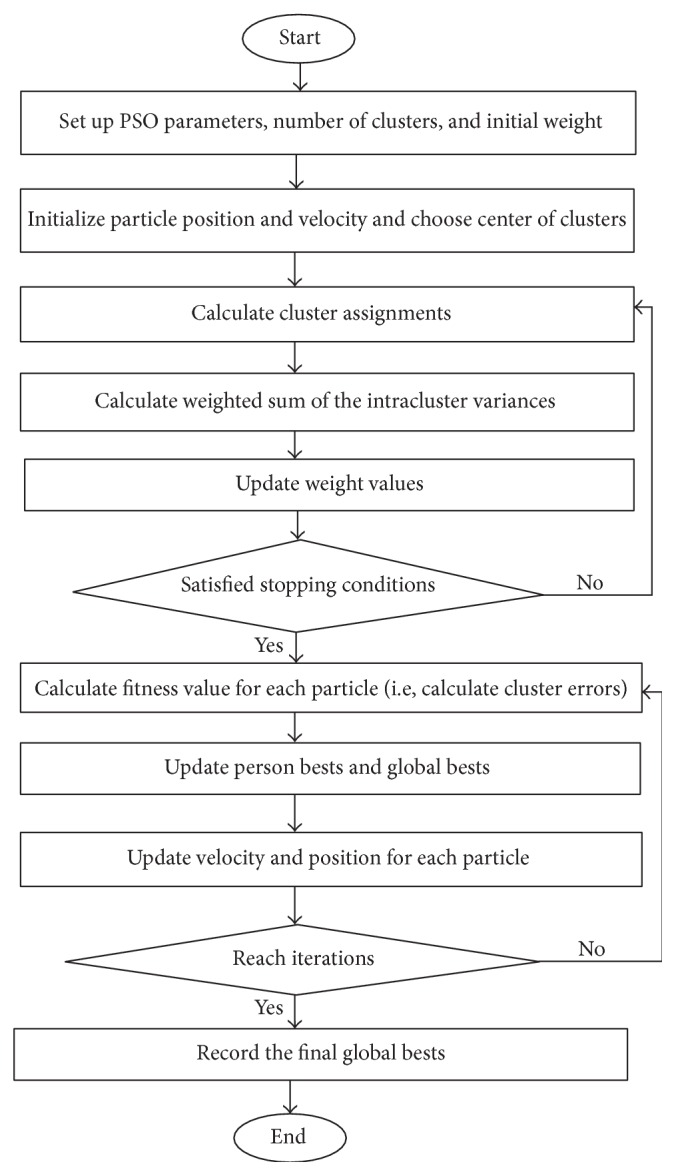
Flowchart of the PSO MinMax *k*-means algorithm.

**Table 1:** Comparative results in different parameter value on the Pendigit data set.

Value of parameter	*E*_max_	*E*_sum_
*p*_max_ = 0.5, *β* = 0.3	8510	62094
*p*_max_ = 0.4, *β* = 0.3	7609	61184
*p*_max_ = 0.3, *β* = 0.3	7948	60993
*p*_max_ = 0.5, *β* = 0.1	16826	71546
*p*_max_ = 0.4, *β* = 0.1	10394	63285
*p*_max_ = 0.3, *β* = 0.1	7918	60993
*p*_max_ = 0.5, *β* = 0	7744	61116
*p*_max_ = 0.4, *β* = 0	7740	61100
*p*_max_ = 0.3, *β* = 0	7924	60994

**Table 2:** Comparative results on *S*_1_ data set.

Method	Initial State 1		Initial State 2		Initial State 3		Initial State 4		Initial State 5		Mean error	
	*E*_max_	*E*_sum_	*E*_max_	*E*_sum_	*E*_max_	*E*_sum_	*E*_max_	*E*_sum_	*E*_max_	*E*_sum_	*E*_max_	*E*_sum_
*k*-means	90.8431	**329.4133**	90.8431	329.4258	90.8431	329.4258	90.8431	329.4258	90.8431	**329.4133**	90.8431	329.4208
MinMax (*p*_max_ = 0.5, *β* = 0.3)	**87.1170**	329.6677	**87.1170**	329.6677	**87.1170**	329.6677	**87.1170**	329.6677	**87.1170**	329.6677	**87.1170**	329.6677
MinMax (*p*_max_ = 0.5, *β* = 0.1)	**87.1170**	329.6677	**87.1170**	329.6677	**87.1170**	329.6677	**87.1170**	329.6677	**87.1170**	329.6677	**87.1170**	329.6677
MinMax (*p*_max_ = 0.5, *β* = 0)	**87.1170**	329.6352	**87.1170**	329.6352	**87.1170**	329.6352	**87.1170**	329.6352	**87.1170**	329.6352	**87.1170**	329.6352
MinMax (*p*_max_ = 0.3, *β* = 0.3)	89.3927	329.4572	**87.1170**	329.5055	**87.1170**	329.5055	89.3927	329.4572	89.3927	329.4572	88.4824	329.4765
MinMax (*p*_max_ = 0.3, *β* = 0.1)	89.3927	329.4766	**87.1170**	329.5055	**87.1170**	329.5055	89.3927	329.4572	89.3927	329.4572	88.4824	329.4804
MinMax (*p*_max_ = 0.3, *β* = 0)	89.3927	329.4572	**87.1170**	329.5055	**87.1170**	329.5055	89.3927	329.4572	89.3927	329.4572	88.4824	329.4765
MinMax (*p*_max_ = 0.1, *β* = 0.3)	90.0813	329.4188	88.5673	329.4616	88.5673	329.4616	90.8431	**329.4133**	90.0813	329.4188	89.6281	329.4348
MinMax (*p*_max_ = 0.1, *β* = 0.1)	90.0813	329.4188	88.5673	329.4616	88.5673	329.4616	90.8431	**329.4133**	90.0813	329.4188	89.6281	329.4348
MinMax (*p*_max_ = 0.1, *β* = 0)	90.0813	329.4188	88.5673	329.4616	88.5673	329.4616	90.8431	**329.4133**	90.0813	329.4188	89.6281	329.4348
MinMax (*p*_max_ = 0, *β* = 0)	90.8431	**329.4133**	90.8431	329.4258	90.8431	329.4258	90.8431	329.4258	90.8431	**329.4133**	90.8431	329.4208
PSO MinMax	90.8431	**329.4133**	90.8431	**329.4133**	90.8431	**329.4133**	90.0813	**329.4133**	90.8431	**329.4133**	90.8431	**329.4133**
PSO MinMax	**87.1170**	329.6207	**87.1170**	329.5857	**87.1170**	329.5857	**87.1170**	329.4833	**87.1170**	329.5857	**87.1170**	329.5722

**Table 3:** Comparative results on *S*_2_ data set.

Method	Initial State 1		Initial State 2		Initial State 3		Initial State 4		Initial State 5		Mean error	
	*E*_max_	*E*_sum_	*E*_max_	*E*_sum_	*E*_max_	*E*_sum_	*E*_max_	*E*_sum_	*E*_max_	*E*_sum_	*E*_max_	*E*_sum_
*k*-means	62.5878	**105.5999**	62.5878	**105.5999**	62.5878	**105.5999**	62.5878	**105.5999**	62.5878	**105.5999**	62.5878	**105.5999**
MinMax (*p*_max_ = 0.5, *β* = 0.3)	54.0427	109.0927	54.0427	109.0927	54.0427	109.0927	54.0427	109.0927	54.0427	109.0927	54.0427	109.0927
MinMax (*p*_max_ = 0.5, *β* = 0.1)	54.0464	109.1226	54.0464	109.1226	54.0464	109.1226	54.0464	109.1226	54.0464	109.1226	54.0464	109.1226
MinMax (*p*_max_ = 0.5, *β* = 0)	54.0464	109.1226	54.0464	109.1226	54.0464	109.1226	54.0464	109.1226	54.0464	109.1226	54.0464	109.1226
MinMax (*p*_max_ = 0.3, *β* = 0.3)	57.3660	106.6937	57.3660	106.6937	57.3660	106.6937	57.3660	106.6937	57.3660	106.6937	57.3660	106.6937
MinMax (*p*_max_ = 0.3, *β* = 0.1)	57.3660	106.6937	57.3660	106.6937	57.3660	106.6937	57.3660	106.6937	57.3660	106.6937	57.3660	106.6937
MinMax (*p*_max_ = 0.3, *β* = 0)	57.3660	106.6937	57.3660	106.6937	57.3660	106.6937	57.3660	106.6937	57.3660	106.6937	57.3660	106.6937
MinMax (*p*_max_ = 0.1, *β* = 0.3)	61.0903	105.6490	61.0903	105.6490	61.0903	105.6490	61.0903	105.6490	61.0903	105.6490	61.0903	105.6490
MinMax (*p*_max_ = 0.1, *β* = 0.1)	61.0903	105.6490	61.0903	105.6490	61.0903	105.6490	61.0903	105.6490	61.0903	105.6490	61.0903	105.6490
MinMax (*p*_max_ = 0.1, *β* = 0)	61.0903	105.6490	61.0903	105.6490	61.0903	105.6490	61.0903	105.6490	61.0903	105.6490	61.0903	105.6490
MinMax (*p*_max_ = 0, *β* = 0)	62.5878	**105.5999**	62.5878	**105.5999**	62.5878	**105.5999**	62.5878	**105.5999**	62.5878	**105.5999**	62.5878	**105.5999**
PSO MinMax	62.5878	**105.5999**	62.5878	**105.5999**	62.5878	**105.5999**	62.5878	**105.5999**	62.5878	**105.5999**	62.5878	**105.5999**
PSO MinMax	**52.1071**	110.8688	**52.1071**	110.8688	**52.1071**	110.8688	**52.1071**	110.8688	**52.1071**	110.8688	52.1071	**110.8688**

**Table 4:** The brief description of the real data sets.

Data set	Instances	Attributes	Classes	Balanced
Coil2	216	1000	3	Yes
Yeast	1350	8	5	No
Pendigits	10992	16	10	Almost
Ecoli	307	7	4	No

**Table 5:** Comparative results on Coil2 data set.

Method	Initial State 1		Initial State 2		Initial State 3		Initial State 4		Initial State 5		Mean error	
	*E*_max_	*E*_sum_	*E*_max_	*E*_sum_	*E*_max_	*E*_sum_	*E*_max_	*E*_sum_	*E*_max_	*E*_sum_	*E*_max_	*E*_sum_
*k*-means	58.8338	**154.0274**	58.8338	**154.0274**	78.2208	156.6057	68.2271	156.9333	122.1251	158.1630	77.2501	155.9514
MinMax (*p*_max_ = 0.5, *β* = 0.3)	58.8645	154.1049	58.8645	154.1049	58.8645	154.1049	58.8645	154.1049	59.0939	156.5141	58.9104	154.5867
MinMax (*p*_max_ = 0.5, *β* = 0.1)	57.0210	154.7626	**57.0210**	154.7626	**57.0210**	154.7626	**57.0210**	154.7626	**57.0210**	154.7626	**57.0210**	154.7626
MinMax (*p*_max_ = 0.5, *β* = 0)	58.8645	154.1049	58.8645	154.1049	58.8645	154.1049	58.8645	154.1049	58.8645	154.1049	58.8645	154.1049
MinMax (*p*_max_ = 0.3, *β* = 0.3)	58.8338	**154.0274**	58.8338	**154.0274**	58.8338	**154.0274**	58.8338	154.0274	61.6945	156.3954	59.4061	154.5010
MinMax (*p*_max_ = 0.3, *β* = 0.1)	58.8338	**154.0274**	58.8338	**154.0274**	58.8338	**154.0274**	58.8338	154.0274	58.8338	**154.0274**	58.8338	154.0274
MinMax (*p*_max_ = 0.3, *β* = 0)	58.8338	**154.0274**	58.8338	**154.0274**	58.8338	**154.0274**	58.8338	154.0274	58.8338	**154.0274**	58.8338	154.0274
MinMax (*p*_max_ = 0.1, *β* = 0.3)	58.8338	**154.0274**	58.8338	**154.0274**	58.8338	**154.0274**	77.6769	153.8029	122.1251	158.3375	75.2607	154.8445
MinMax (*p*_max_ = 0.1, *β* = 0.1)	58.8338	**154.0274**	58.8338	**154.0274**	58.8338	**154.0274**	77.6769	153.8029	122.1251	158.6074	75.2607	154.8985
MinMax (*p*_max_ = 0.1, *β* = 0)	58.8338	**154.0274**	58.8338	**154.0274**	78.2208	156.6057	77.6769	153.8029	122.1251	158.6074	79.1381	155.4142
MinMax (*p*_max_ = 0, *β* = 0)	58.8338	**154.0274**	58.8338	**154.0274**	78.2208	156.6057	68.2271	156.9333	122.1251	158.1630	77.2501	155.9514
PSO MinMax	58.8338	**154.0274**	58.8338	**154.0274**	58.8338	**154.0274**	78.5526	**153.7484**	58.8338	**154.0274**	62.7816	**153.9716**
PSO MinMax	**56.9911**	154.6846	58.8338	**154.0274**	58.8338	**154.0274**	58.8338	154.0274	58.8338	**154.0274**	58.4653	154.1588

**Table 6 tab6:** Comparative results on Yeast data set.

Method	Initial State 1		Initial State 2		Initial State 3		Initial State 4		Initial State 5		Mean error	
	*E*_max_	*E*_sum_	*E*_max_	*E*_sum_	*E*_max_	*E*_sum_	*E*_max_	*E*_sum_	*E*_max_	*E*_sum_	*E*_max_	*E*_sum_
*k*-means	11.8980	**51.1611**	13.6188	50.9920	19.4485	53.5283	13.6188	50.9920	13.5837	50.9907	14.4336	51.5328
MinMax (*p*_max_ = 0.5, *β* = 0.3)	16.0624	53.8103	11.1672	51.3847	16.0624	53.8103	11.1672	51.3847	11.0701	51.3795	13.1059	52.3539
MinMax (*p*_max_ = 0.5, *β* = 0.1)	10.6440	51.3867	21.1602	64.8241	10.6440	51.3867	21.1602	64.8241	11.0701	51.3795	14.9357	56.7602
MinMax (*p*_max_ = 0.5, *β* = 0)	10.7905	51.3745	12.0426	51.2481	10.9650	51.3287	12.0426	51.2481	11.6988	51.2497	11.5079	51.2898
MinMax (*p*_max_ = 0.3, *β* = 0.3)	10.9719	51.2913	11.6917	51.2680	11.0223	51.2958	11.6917	51.2720	11.6810	51.2268	11.4117	51.2708
MinMax (*p*_max_ = 0.3, *β* = 0.1)	11.0067	51.2912	12.0105	51.2482	11.0565	51.2899	12.0105	51.2482	11.6810	51.2268	11.5530	51.2609
MinMax (*p*_max_ = 0.3, *β* = 0)	10.9719	51.2913	11.9915	51.2482	11.0223	51.2958	11.9915	51.2482	11.6810	51.2268	11.5316	55.2621
MinMax (*p*_max_ = 0.1, *β* = 0.3)	11.4906	51.2024	12.6413	51.1240	11.5863	51.1879	12.7464	51.0934	12.7564	51.0206	12.2442	51.1260
MinMax (*p*_max_ = 0.1, *β* = 0.1)	11.4906	51.2024	12.6869	51.1236	11.5958	51.1879	12.7505	51.0962	12.7564	51.0206	12.2560	51.1261
MinMax (*p*_max_ = 0.1, *β* = 0)	11.4906	51.2024	12.6869	51.1236	11.5958	51.1913	12.7505	51.0962	12.7564	51.0206	12.2560	51.1261
MinMax (*p*_max_ = 0, *β* = 0)	11.8980	**51.1611**	13.6188	50.9920	19.4485	53.5283	13.6188	50.9920	13.5837	50.9907	14.4336	51.5328
PSO MinMax	11.8980	**51.1611**	13.0750	**50.9871**	13.3736	**50.9891**	13.0750	**50.9871**	13.1598	**50.9869**	12.9163	**51.0233**
PSO MinMax	**10.6149**	51.5056	**10.6360**	51.3925	**10.5965**	51.4087	**10.6205**	51.4043	**10.9874**	51.4089	**10.6911**	51.4240

**Table 7 tab7:** Comparative results on Pendigit data set.

Method	Initial State 1		Initial State 2		Initial State 3		Initial State 4		Initial State 5		Mean error	
	*E*_max_	*E*_sum_	*E*_max_	*E*_sum_	*E*_max_	*E*_sum_	*E*_max_	*E*_sum_	*E*_max_	*E*_sum_	*E*_max_	*E*_sum_
*k*-means	20443	67183	15171	61478	14322	**59899**	7839	61733	12262	**59628**	14007	61984
MinMax (*p*_max_ = 0.5, *β* = 0.3)	8969	63634	9160	62233	8955	61767	7311	61911	10951	63265	9096	62562
MinMax (*p*_max_ = 0.5, *β* = 0.1)	7355	61874	9389	62097	15654	73892	23296	77247	26142	75272	16367	70076
MinMax (*p*_max_ = 0.5, *β* = 0)	7480	61792	9779	61903	10085	60836	7531	61839	6891	60234	8353	61320
MinMax (*p*_max_ = 0.3, *β* = 0.3)	10814	61554	10842	61474	10463	60617	7600	61809	6994	60181	9342	61127
MinMax (*p*_max_ = 0.3, *β* = 0.1)	7599	61737	10842	61475	10474	60611	7600	61809	6994	60179	8701	61162
MinMax (*p*_max_ = 0.3, *β* = 0)	7599	61737	10842	61475	10474	60611	7600	61809	6994	60179	8701	61162
MinMax (*p*_max_ = 0.1, *β* = 0.3)	13231	60986	13235	60924	12521	59990	7736	61740	11434	59713	11631	60670
MinMax (*p*_max_ = 0.1, *β* = 0.1)	11041	61520	13235	60924	12542	**59989**	7736	61740	11434	59713	11197	60777
MinMax (*p*_max_ = 0.1, *β* = 0)	13231	60986	13235	60924	12542	**59989**	7736	61740	11434	59713	11636	60670
MinMax (*p*_max_ = 0, *β* = 0)	20443	67183	15171	61478	14322	**59899**	7839	61733	12262	**59628**	14007	61984
PSO MinMax	7319	**60047**	13394	**60907**	14322	**59899**	7296	**60055**	12262	**59628**	10919	**60107**
PSO MinMax	**6678**	60393	**6887**	61424	**8491**	63718	**6672**	60396	**6669**	60393	**7079**	61265

**Table 8:** Comparative results on Ecoli data set.

Method	Initial State 1		Initial State 2		Initial State 3		Initial State 4		Initial State 5		Mean error	
	*E*_max_	*E*_sum_	*E*_max_	*E*_sum_	*E*_max_	*E*_sum_	*E*_max_	*E*_sum_	*E*_max_	*E*_sum_	*E*_max_	*E*_sum_
*k*-means	6.7989	**15.3683**	7.1814	15.3672	6.0589	15.7607	5.6841	15.7828	6.7989	15.3683	6.5039	15.5294
MinMax (*p*_max_ = 0.5, *β* = 0.3)	**4.7952**	15.7294	**4.7952**	15.7294	**4.7952**	15.7294	**4.7952**	15.7294	**4.7952**	15.7294	**4.7952**	15.7294
MinMax (*p*_max_ = 0.5, *β* = 0.1)	5.2944	15.7008	5.2944	15.7008	**4.7952**	**15.7281**	5.0384	15.8646	5.2944	15.7008	5.1434	15.7390
MinMax (*p*_max_ = 0.5, *β* = 0)	5.4347	16.1918	5.4347	16.1918	5.1738	15.7109	5.1738	15.7109	5.4347	16.1918	5.3303	15.9994
MinMax (*p*_max_ = 0.3, *β* = 0.3)	5.5362	15.4852	5.5362	15.4852	5.6841	15.7924	5.4652	15.8300	5.5362	15.4852	5.5516	15.6156
MinMax (*p*_max_ = 0.3, *β* = 0.1)	5.5362	15.4852	5.5362	15.4852	5.6841	15.7924	5.4652	15.8300	5.5362	15.4852	5.5516	15.6156
MinMax (*p*_max_ = 0.3, *β* = 0)	5.5362	15.4852	5.5362	15.4852	5.6841	15.7924	5.4652	15.8300	5.5362	15.4852	5.5516	15.6156
MinMax (*p*_max_ = 0.1, *β* = 0.3)	6.2941	15.3943	6.7989	15.3683	6.0589	15.7607	5.6841	15.7822	6.7989	**15.3683**	6.3270	15.5348
MinMax (*p*_max_ = 0.1, *β* = 0.1)	5.8672	15.4332	6.7989	15.3683	6.0589	15.7607	5.6841	15.7822	6.7989	**15.3683**	6.2416	15.5425
MinMax (*p*_max_ = 0.1, *β* = 0)	5.8672	15.4332	6.7989	15.3683	6.0589	15.7607	5.6841	15.7822	6.7989	**15.3683**	6.2416	15.5425
MinMax (*p*_max_ = 0, *β* = 0)	6.7989	**15.3683**	7.1814	15.3672	6.0589	15.7607	5.6841	15.7828	6.7989	**15.3683**	6.5039	15.5294
PSO MinMax	6.7989	**15.3683**	6.9020	**15.3664**	**4.7952**	**15.7281**	5.6920	**15.4645**	6.7989	**15.3683**	6.1974	**15.4591**
PSO MinMax	**4.7952**	15.7281	**4.7952**	15.7294	**4.7952**	15.7294	**4.7952**	15.7294	**4.7952**	15.7281	**4.7952**	15.7289

**Table 9:** Value of parameters in PSO MinMax algorithm.

Data set	State 1		State 2		State 3		State 4		State 5	
	*p*	*β*	*p*	*β*	*p*	*β*	*p*	*β*	*p*	*β*
*S*_1_	0	0.3986	0.0342	0.1352	0.0350	0.4710	0.0331	0.2876	0.0331	0.2876
	0.4052	0.1871	0.3889	0.2407	0.3712	0.1194	0.2882	0.1000	0.3624	0.3775

*S*_2_	0.0091	0.0076	0.0088	0.4151	0	0.4034	0	0.4069	0.0116	0.0604
	0.5000	0.2351	0.5000	0.1176	0.5000	0.0844	0.5000	0.2352	0.5000	0.2933

Coil2	0.4382	0.2597	0.3283	0.4405	0.0570	0.0098	0.0864	0.3274	0.4067	0.4891
	0.5000	0.3370	0.3283	0.4405	0.0570	0.0098	0.4079	0.3010	0.4067	0.4891

Yeast	0.0570	0.0098	0.0284	0.4602	0.0081	0.1661	0.0307	0.3009	0.0204	0.3449
	0.4906	0.4870	0.4994	0.5000	0.4906	0.4030	0.5000	0.5000	0.5000	0.5000

Pendigit	0.1862	0	0.0852	0.3622	0	0.4125	0.1900	0.0446	0	0.3126
	0.5000	0.3996	0.2754	0.0315	0.5000	0.2609	0.5000	0.2609	0.5000	0.4477

Ecoli	0	0.0793	0.0536	0.3177	0.4262	0.1783	0.1898	0.0506	0.0888	0.2523
	0.4773	0.2823	0.4848	0.4448	0.4756	0.3074	0.4213	0.1610	0.4890	0.4521

## References

[1] Xu R., Wunsch D. C. (2005). Survey of clustering algorithms. *IEEE Transactions on Neural Networks*.

[2] Jain A. K. (2010). Data clustering: 50 years beyond K-means. *Pattern Recognition Letters*.

[3] Celebi M. E., Kingravi H. A., Vela P. A. (2013). A comparative study of efficient initialization methods for the k-means clustering algorithm. *Expert Systems with Applications*.

[4] Peña J. M., Lozano J. A., Larrañaga P. (1999). An empirical comparison of four initialization methods for the K-Means algorithm. *Pattern Recognition Letters*.

[5] Likas A., Vlassis N., Verbeek J. J. (2003). The global k-means clustering algorithm. *Pattern Recognition*.

[6] Bagirov A. M. (2008). Modified global k-means algorithm for minimum sum-of-squares clustering problems. *Pattern Recognition*.

[7] Bagirov A. M., Ugon J., Webb D. (2011). Fast modified global k-means algorithm for incremental cluster construction. *Pattern Recognition*.

[8] Tzortzis G. F., Likas A. C. (2009). The global kernel *k*-means algorithm for clustering in feature space. *IEEE Transactions on Neural Networks*.

[9] Tzortzis G., Likas A. The global kernel k-means clustering algorithm.

[10] Zang X., Vista F. P., Chong K. T. (2014). Fast global kernel fuzzy c-means clustering algorithm for consonant/vowel segmentation of speech signal. *Journal of Zhejiang University-Science C (Computers & Electronics)*.

[11] Khan S. S., Ahmad A. (2004). Cluster center initialization algorithm for *K*-means clustering. *Pattern Recognition Letters*.

[12] Murty M. N., Jain A. K., Flynn P. J. (1999). Data clustering: a review. *ACM Computing Surveys*.

[13] Arthur D., Vassilvitskii S. *k*-means++: the advantages of careful seeding.

[14] Banerjee A., Ghosh J. (2004). Frequency-sensitive competitive learning for scalable balanced clustering on high-dimensional hyperspheres. *IEEE Transactions on Neural Networks*.

[15] Tzortzis G., Likas A. (2014). The MinMax *k*-Means clustering algorithm. *Pattern Recognition*.

[16] Eslamnezhad M., Varjani A. Y. Intrusion detection based on MinMax K-means clustering.

[17] Eberhart R., Kennedy J. A new optimizer using particle swarm theory.

[18] Salerno J. Using the particle swarm optimization technique to train a recurrent neural model.

[19] Zhang C., Shao H., Li Y. Particle swarm optimization for evolving artificial neural network.

[20] Lu J., Hu H., Bai Y. (2015). Generalized radial basis function neural network based on an improved dynamic particle swarm optimization and AdaBoost algorithm. *Neurocomputing*.

[21] Xia W., Wu Z., Zhang W., Yang G. A new hybrid optimization algorithm for the job-shop scheduling problem.

[22] Koay C. A., Srinivasan D. Particle swarm optimization-based approach for generator maintenance scheduling.

[23] Wu B., Zhao Y., Ma Y., Dong H., Wang W. Particle swarm optimization method for vehicle routing problem.

[24] Jiang W., Zhang Y., Xie J. A particle swarm optimization algorithm with crossover for vehicle routing problem with time windows.

[25] Karami A., Guerrero-Zapata M. (2015). A fuzzy anomaly detection system based on hybrid PSO-Kmeans algorithm in content-centric networks. *Neurocomputing*.

[26] Chen C. Y., Ye F. Particle swarm optimization algorithm and its application to clustering analysis.

[27] Esmin A. A. A., Pereira D. L., de Araújo F. P. A. Study of different approach to clustering data by using the particle swarm optimization algorithm.

[28] Paterlini S., Krink T. (2006). Differential evolution and particle swarm optimisation in partitional clustering. *Computational Statistics & Data Analysis*.

[29] Van der Merwe D. W., Engelbrecht A. P. Data clustering using particle swarm optimization.

[30] Rana S., Jasola S., Kumar R. (2011). A review on particle swarm optimization algorithms and their applications to data clustering. *Artificial Intelligence Review*.

[31] Ahmadyfard A., Modares H. Combining PSO and k-means to enhance data clustering.

[32] Kuo R. J., Wang M. J., Huang T. W. (2011). An application of particle swarm optimization algorithm to clustering analysis. *Soft Computing*.

[33] Shi Y., Eberhart R. A modified particle swarm optimizer.

[34] Shi Y., Eberhart R. (1998). Parameter selection in particle swarm optimization. *Evolutionary programming VII: Proceedings of EP '98*.

[35] Shi Y., Eberhart R. C. Empirical study of particle swarm optimization.

[36] Chatterjee A., Siarry P. (2006). Nonlinear inertia weight variation for dynamic adaptation in particle swarm optimization. *Computers and Operations Research*.

[37] Lei K., Qiu Y., He Y. A new adaptive well-chosen inertia weight strategy to automatically harmonize global and local search ability in particle swarm optimization.

[38] Clerc M. The swarm and the queen: towards a deterministic and adaptive particle swarm optimization.

[39] Chan C.-L., Chen C.-L. (2015). A cautious PSO with conditional random. *Expert Systems with Applications*.

[40] Fang C., Jin W., Ma J. (2013). k′-Means algorithms for clustering analysis with frequency sensitive discrepancy metrics. *Pattern Recognition Letters*.

[41] Nene S. A., Nayar S. K., Murase H. (1996). Columbia object image library (COIL-20).

[42] Frank A., Asuncion A. (2010). *UCI Machine Learning Repository*.

